# A TLR2/S100A9/CXCL-2 signaling network is necessary for neutrophil recruitment in acute and chronic liver injury in the mouse

**DOI:** 10.1016/j.jhep.2013.12.005

**Published:** 2014-04

**Authors:** Anna Moles, Lindsay Murphy, Caroline L. Wilson, Jayashree Bagchi Chakraborty, Christopher Fox, Eek Joong Park, Jelena Mann, Fiona Oakley, Rachel Howarth, John Brain, Steven Masson, Michael Karin, Ekihiro Seki, Derek A. Mann

**Affiliations:** 1 Fibrosis Research Group, Institute of Cellular Medicine, Newcastle University, Newcastle Upon Tyne, UK; 2 Division of Gastroenterology, Department of Medicine, University of California, San Diego, School of Medicine, La Jolla, CA, USA; 3 Department of Pharmacology and Pathology Laboratory of Gene Regulation and Signal Transduction, School of Medicine, University of California, San Diego, 9500 Gilman Drive, La Jolla, CA 92093-0723, USA

**Keywords:** APAP ALF, acetaminophen acute liver failure, AHH, acute alcoholic hepatitis, α-SMA, α-smooth muscle actin, ALT, alanine transaminase, ALD, alcoholic liver disease, CCl_4_, carbon tetrachloride, CCL-2, chemokine (C-C motif) ligand 2, CCL-5, chemokine (C-C motif) ligand 5, CXCL-2, chemokine (C-X-C motif) ligand 2, CXCL-1, chemokine (C-X-C motif) ligand 1, IHC, immunohistochemistry, IL6, interleukin-6, LTA, lipoteichoic acid, NASH, non-alcoholic steatohepatitis, PBC, primary biliary cirrhosis, PSC, primary sclerosing cholangitis, PCNA, proliferating cell nuclear antigen, ROS, reactive oxygen species, S100A8, S100 calcium binding protein A8, S100A9, S100 calcium binding protein A9, TLR, toll like receptor, TNF-α, tumor necrosis factor-α, Liver fibrosis, Neutrophil, Toll like receptor, Inflammation, S100A9

## Abstract

**Background & Aims:**

Neutrophils are important immune effectors required for sterile and non-sterile inflammatory responses. However, neutrophils are associated with pathology in drug-induced liver injury, acute alcoholic liver disease, and ischemia-reperfusion injury. An understanding of the complex mechanisms that control neutrophil recruitment to the injured liver is desirable for developing strategies aimed at limiting neutrophil-mediated cellular damage.

**Methods:**

Wt, *tlr2*^−/−^, *tlr4*^−/−^, and *s100a9*^−/−^ mice were administered CCl_4_ either acutely (8, 24, 48, or 72 h) or chronically (8 weeks) and livers investigated by histological (IHC for neutrophils, fibrogenesis, proliferation, and chemotactic proteins) or molecular approaches (qRT-PCR for neutrophil chemoattractant chemokines and cytokines as well as pro-fibrogenic genes).

**Results:**

Mice lacking TLR2 or S100A9 failed to recruit neutrophils to the injured liver and had a defective hepatic induction of the neutrophil chemokine CXCL-2. Hierarchy between TLR2 and S100A9 proved to be complex. While induction of S100A9 was dependent on TLR2 in isolated neutrophils, there was a more complicated two-way signalling cross-talk between TLR2 and S100A9 in whole liver. However, wound-healing and regenerative responses of the liver were unaffected in these genetic backgrounds as well as in wild type mice, in which neutrophils were depleted by infusion of Ly-6G antibody.

**Conclusions:**

We have identified TLR2 and S100A8/S100A9 as key regulators of hepatic CXCL-2 expression and neutrophil recruitment. This novel TLR2-S100A9-CXCL-2 pathway may be of use in development of new strategies for selectively manipulating neutrophils in liver disease without impairing normal wound healing and regenerative responses.

## Introduction

Hepatic infiltration by neutrophils and their subsequent activation are a rapid response to sterile and non-sterile tissue injury [1]. Following extravasation into the parenchyma, neutrophils directly interact with hepatocytes via their surface LFA-1 (CD11b/CD18) and Mac-1 receptors. Engagement of the latter stimulates production of reactive oxygen species (ROS), which is important for host-defence but can be cytotoxic; neutrophil-derived ROS can diffuse into adjacent hepatocytes and either directly induce cellular damage or more likely cause mitochondrial dysfunction and necrosis [2]. Additionally degranulation of neutrophils results in the release of proteases that can contribute to hepatocellular damage and death [3]. Clinical conditions, with evidence for neutrophil activation and hepatocellular death, include alcoholic hepatitis [4], acetaminophen (APAP)-induced acute liver injury [2], and ischemia-reperfusion injury [5]. The extent of neutrophil infiltration in alcoholic hepatitis is correlated with disease severity [6]. There is experimental evidence for neutrophil-mediated cell death in liver injury models including acute alcoholic hepatitis [7]. By contrast, a role for neutrophils in chronic liver disease is less clear. Cirrhosis is associated with elevated levels of circulating and hepatic neutrophil chemokines such as IL-8, while hepatic neutrophils are also described to be a feature of progressive fibrotic disease [8]. However, experimental models of biliary liver disease suggest a minor if any functional contribution of neutrophils to fibrogenesis [9]. But it remains likely that persistence of activated neutrophils in chronic liver disease is contributory to disease progression and outcome, including hepatocellular carcinoma [10]. As such, a better understanding of the regulation of neutrophil recruitment is important, as is the identification of molecular targets that might be exploited for therapeutic manipulation of neutrophils in diseased tissues.

McDonald *et al.* reported that a dynamic multistep network of directional cues serves to recruit neutrophils to sites of cellular damage under sterile conditions. At least three key mechanisms were identified: (i) Activation of the Nlrp3 inflammasome by ATP released from necrotic cells, which promoted adherence of circulating neutrophils to sinusoids, (ii) generation of a chemokine gradient directing neutrophils to the site of cellular damage, and (iii) formyl-peptide signals from necrotic cells that help guide neutrophils through the sinusoids to the site of injury [11]. Activation of Toll-like-receptors (TLRs) also facilitates neutrophil recruitment and recent studies have highlighted the increased expression and functional importance of TLR2, 4, and 9 in acute and chronic liver disease [12].

Here we have used a mouse gene knockout approach to investigate the role of TLR2 in the response to toxic (carbon tetrachloride, CCl_4_) liver injury. We demonstrate a critical role for TLR2 (but not TLR4) for recruitment of neutrophils to the injured liver. TLR2 was required for expression of the CXCL-1 (*KC*) CXCL-2 (*MIP-2*) neutrophil chemokines by hepatic macrophages. In addition, the myeloid-related proteins MRP-8 (*S100A8*) and MRP-14 (*S100A9*) were also induced in response to liver injury and were responsive to TLR2 activation. Under normal physiological conditions *S100A8* and *S100A9* are expressed in neutrophils, monocytes, and eosinophils [13], but following tissue damage they are induced in epithelial and endothelial cells and upon secretion act as powerful leukocyte chemoattractants [14]. S100A8 and S100A9 form functional homodimers and heterodimers (the latter known as Calprotectin). Mice lacking S100A9 also lack S100A8 due to instability of the S100A8 protein in the absence of S100A9 protein; hence *s100a9^−/−^* mice fail to express Calprotectin under resting or injury-induced states. We show here that *s100a9^−/−^* mice display a similar phenotype to *tlr2^−/−^* animals with defective induction of hepatic CXCL-2 induction and reduced neutrophil recruitment. However, neither deficiency in TLR2 or S100A9 or antibody-mediated neutrophil depletion had any impact on activation of hepatic stellate cells (HSCs) or wound-repair. Hence, a hepatic TLR2-S100A9-CXCL-2 pathway may be an interesting target for the selective manipulation of neutrophils in acute and chronic liver disease.

## Materials and methods

### *In vivo* models of rodent acute liver injury and fibrogenesis

Wt, *tlr2*^−/−^ [15], *tlr4*^−/−^ [16], and *s100a*9^−/−^ [17] mice were provided by Prof M. Karin, Prof E. Seki and Prof N. Hogg. Single intraperitoneal injection of CCl_4_ at a dose of 2 μl (CCl_4_:olive oil, 1:1 [v:v])/g body weight was administered for 8, 24, 48, and 72 h to 8–10 week old male littermates. 8–10 week old male C57Bl/6 mice were injected with pure LTA at 250 μg/mouse 30 min prior to acute CCl_4_ challenge. Mice were pre-treated with Ly-6G or IgG control antibody for 12 h before LTA injection. Animals were culled at 48 h post-CCl_4_ injection. At least 5 animals were used per treatment group.

### *In vivo* models of rodent chronic liver injury and fibrogenesis

8–10 week old male *s100a*9^−/−^ and wt littermate mice were injected with CCl_4_ intraperitoneally (IP) twice a week at a dose of 2 μl (CCl_4_:olive oil, 1:3, [v:v])/g body weight during 8 weeks or bile duct ligation was performed as previously described for 14 days [18]. For the chronic CCl_4_ model, animals were culled either at 24 h (peak) or 7 days (recovery) after the last CCl_4_ injection. At least 7 animals were used per group of treatment.

### Statistical analysis

Data is expressed as mean ± S.E.M*.* (*standard error*). A minimum of 5 animals per group were used in the experimental animal models. All *p* values were calculated using a two tailed paired Student’s *t* test or a one way ANOVA and *^∗^p* ⩽0.05 or *^∗∗^p* ⩽0.01 was considered statistically significant.

## Results

### Neutrophils are a common feature of human liver disease

**Fig. 1:**
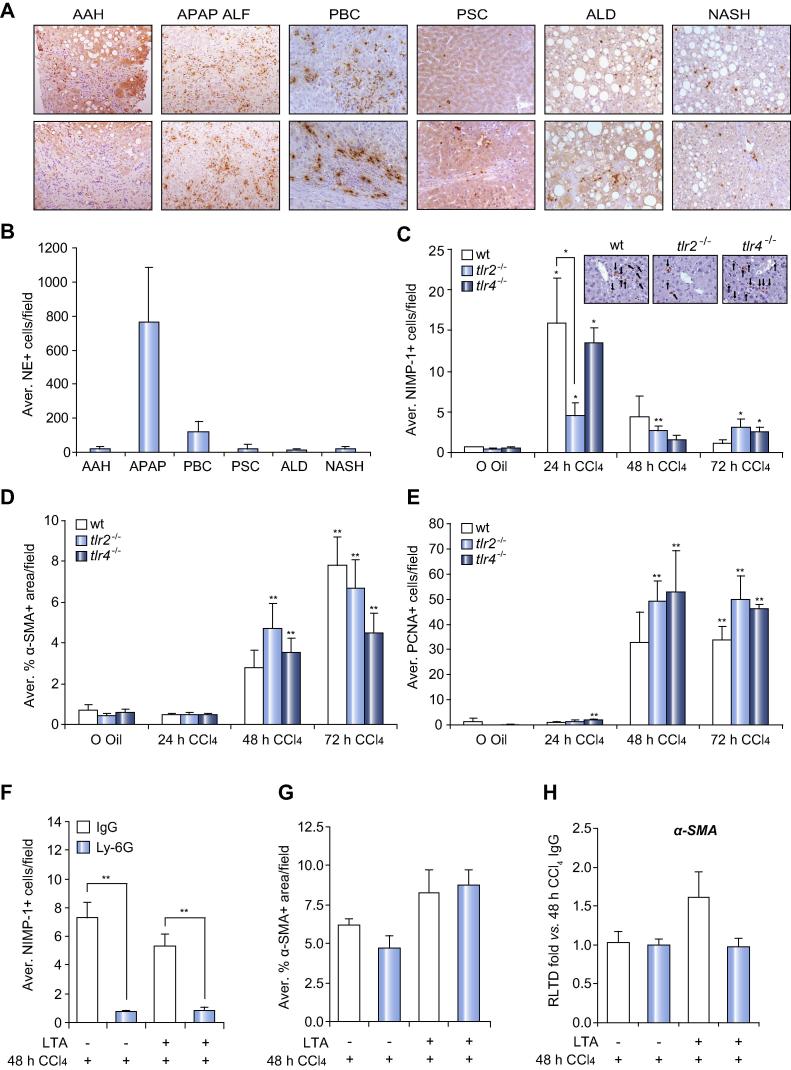
***Tlr2^−/−^* presents deficient neutrophil recruitment with no effect on the wound healing response.** Representative 200× photomicrographs of neutrophil elastase IHC from a minimum of 4 different human biopsies of (A) AAH, APAP ALF, PBC, PSC, ALD, and NASH. (B) Average NE+ cells/100× field. (C) Average NIMP-1+ cells/field and representative pictures at 400×, (D) morphometric analysis of α-SMA+ area/field, (E) average PCNA+ cells/field of wt, *tlr2^−/−^*, *tlr4^−/−^* mice after acute CCl_4_ treatment for 24, 48, 72 h. (F) Average NIMP-1+ cells/ field, (G) morphometric analysis of α-SMA+ area/field and (H) *α-SMA* mRNA expression in liver from wt pre-treated with Ly-6G or IgG for 12 h and then treated with CCl_4_ for 48 h ± LTA. *^∗^p* ⩽0.05; *^∗∗^p* ⩽0.01.

Prior to investigating the role of TLRs in neutrophil recruitment we confirmed that these cells are present in diseased human liver. As shown in representative Neutrophil elastase+ (NE+) stained liver sections (Fig. 1A), neutrophils are present in acute alcoholic hepatitis (AAH), acetaminophen acute liver failure (APAP ALF), primary biliary cirrhosis (PBC), primary sclerosing cholangiatis (PSC), alcoholic liver disease (ALD), and non-alcoholic steatohepatitis (NASH) suggestive of neutrophils being a common feature of the diseased liver irrespective of the cause of the underlying injury. As anticipated APAP was associated with high numbers of hepatic neutrophils relative to chronic liver diseases, of the latter neutrophils were highest in PBC livers (Fig. 1B).

### TLR2 is essential for optimal neutrophil recruitment to the damaged liver

To determine if TLR2 is a regulator of neutrophil recruitment we administered CCl_4_ to *tlr2^−/−^* mice and compared their acute response with wt and *tlr4^−/−^* mice at 24, 48, and 72 h. Serum ALT measurements indicated no significant differences in the degree of hepatic damage between the three phenotypes with the exception of *tlr4^−/−^* at 72 h where there was a trend towards higher levels of damage (Supplementary Fig. 1A). IHC staining (NIMP-1) and counting of neutrophils demonstrated the anticipated appearance of high numbers of hepatic neutrophils at 24 h in wt and *tlr4^−/−^* injured mice (Fig. 1C). By contrast there was a 3-fold lower number of neutrophils in *tlr2^−/−^* livers at this time point. In all genotypes, hepatic neutrophils declined to near base-line levels by 48 h. To assess effects of TLR deletions on the acute fibrogenic and regenerative responses following liver damage we performed morphometric analysis of α-SMA+ myofibroblasts (Fig. 1D) and counted PCNA+ hepatocytes (Fig. 1E) respectively. As expected these IHC markers were elevated at 48 and 72 h but no differences were observed between the three genotypes suggesting normal wound-healing in *tlr2^−/−^* liver. A normal fibrogenic response in *tlr2^−/−^* was confirmed by similar induction of transcripts for α-SMA and Collagen I between the genotypes (Supplementary Fig. 1B–C). We conclude that TLR2 is required for optimal recruitment of neutrophils to the hepatic parenchyma, but is dispensable for subsequent wound-repair/fibrogenesis and regenerative responses. However, as previously published [19,20] neutrophil depletion dramatically reduced APAP-induced liver damage (Supplementary Fig. 2A–C).

Since the role of neutrophils has not been formally addressed in the CCl_4_ injury model, we determined the effects of Ly-6G antibody-mediated depletion of circulating neutrophils on fibrogenesis at the 48 h time point in wt animals. Ly-6G treatment led to a dramatic reduction in numbers of circulating and CCl_4_-induced hepatic neutrophils (Fig. 1F and Supplementary Fig. 3A–C). Specificity was confirmed by lack of effect of Ly-6G on hepatic macrophages (Supplementary Fig. 3D). IHC and mRNA analysis of α-SMA revealed no requirement for neutrophils in the fibrogenic response (Fig. 1G and H). Moreover, treatment of CCl_4_-injured mice with the TLR2 agonist LTA was also without effect on fibrogenesis (Fig. 1G and H).

### TLR2 is required for hepatic induction of neutrophil attractants CXCL-2 and TNF-α

**Fig. 2:**
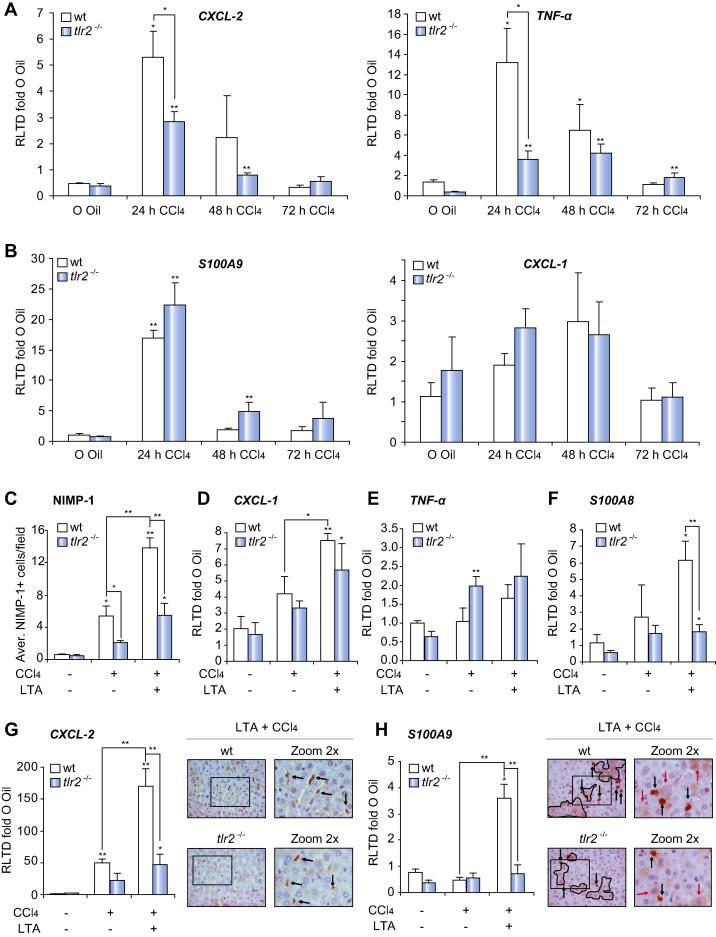
**TLR2 is required for hepatic induction of the neutrophil attractants CXCL-2 and TNF-α.** (A) *CXCL-2*, *TNF-α*, (B) *S100A9*, *CXCL-1* mRNA expression in whole liver of wt, *tlr2^−/−^*, *tlr4^−/−^* mice after acute CCl_4_ treatment for 24, 48, 72 h. (C) Average NIMP-1+ cells/field, (D) *CXCL-1*, (E) *TNF-α*, (F) *S100A8*, (G) *CXCL-2*, and (H) *S100A9* mRNA expression in whole liver and representative 400× pictures of (G) CXCL-2 and (H) S100A9 IHC of wt, and *tlr2^−/−^* after acute CCl_4_ treatment for 8 h ± LTA. *^∗^p* ⩽0.05; *^∗∗^p* ⩽0.01.

Expression *CXCL-1* and *CXCL-2* can be found across a broad range of human liver injuries implicating these chemokines in the recruitment of hepatic neutrophils (Supplementary Fig. 4). The expression of *CXCL-1* and *CXCL-2* and the murine neutrophil chemoattractants *TNF-α* and *S100A9* was examined in CCl_4_ damaged mouse livers. Hepatic *CXCL-2*, *TNF-α*, and *S100A9* transcripts were all robustly induced in wt at 24 h post-CCl_4_ with subsequent decline in their expression (Fig. 2A and B). By contrast, CXCL-1 was only modestly induced and peaked at 48 h (Fig. 2B). Absence of TLR2 was associated with blunted CXCL-2 and TNF-α responses (Fig. 2A), but at this time point had no impact on the induction of *S100A9* (Fig. 2B). ELISA confirmed TLR2 is required for induction of CXCL-2 protein expression (Supplementary Fig. 1D). We next determined the combined effects of LTA and CCl_4_ on hepatic neutrophil chemoattractants comparing wt with *tlr2^−/−^*. For this experiment an earlier 8 h time point was examined, which precedes peak neutrophil accumulation. This time point was associated with a modest increase in numbers of hepatic neutrophils in wt mice which was stimulated 3-fold by co-administration of LTA (Fig. 2C). Neutrophil recruitment at 8 h was blunted in *tlr2^−/−^* mice injured with CCl_4_ and was also reduced in *tlr2^−/−^* mice co-administered LTA and CCl_4_. These data confirm that TLR2 is an important immune trigger for neutrophil recruitment to the liver. Hepatic *CXCL-2* was stimulated by a further 3-fold in mice co-administered LTA and CCl_4_, however this induction was noticeably absent in *tlr2^−/−^* animals (Fig. 2G). IHC analysis confirmed that CXCL-2 was mainly confined to liver macrophages (Fig. 2G). *CXCL-1* (Fig. 2D) and *TNF-α* (Fig. 2E) were only modestly induced at 8 h and no dramatic differences in their expression were noticeable between wt and *tlr2^−/−^* genotypes. Measurement of *S100A8* and *S100A9* transcripts revealed no significant changes at 8 h post-CCl_4_. However, co-administration of LTA and CCl_4_ was associated with elevated neutrophil recruitment (Fig. 2C and G) and with CXCL-2 induced *S100A8* and *S100A9* expression by 6- and 4-fold respectively (Fig. 2F and H). This latter effect was only observed in wt indicating that under these conditions TLR2 was required for induction of Calprotectin. This result contrasted with our earlier observation (Fig. 2B) where CCl_4_ alone at 24 h post-injury was associated with TLR2-independent induction of *S100A9* (Fig. 2B). We therefore confirmed the potential for TLR2 activation to stimulate *S100A9* by treating cultured *ex vivo* neutrophils with LTA. This treatment increased S100A9 expression at the protein level and could be blocked by incubation of neutrophils with anti-TLR2 antibody (Supplementary Fig. 5A). Phosphorylation of P38 was monitored as a positive assay control. As expected, P38 phosphorylation was successfully blocked by an anti-TLR2 antibody after LTA treatment (Supplementary Fig. 5B). IHC analysis confirmed that liver injury was associated with *de novo* induced expression of *S100A9* in hepatocytes in addition to the anticipated expression in neutrophils (Fig. 2H).

### Induction of neutrophil recruitment and hepatic CXCL-2 requires Calprotectin

**Fig. 3:**
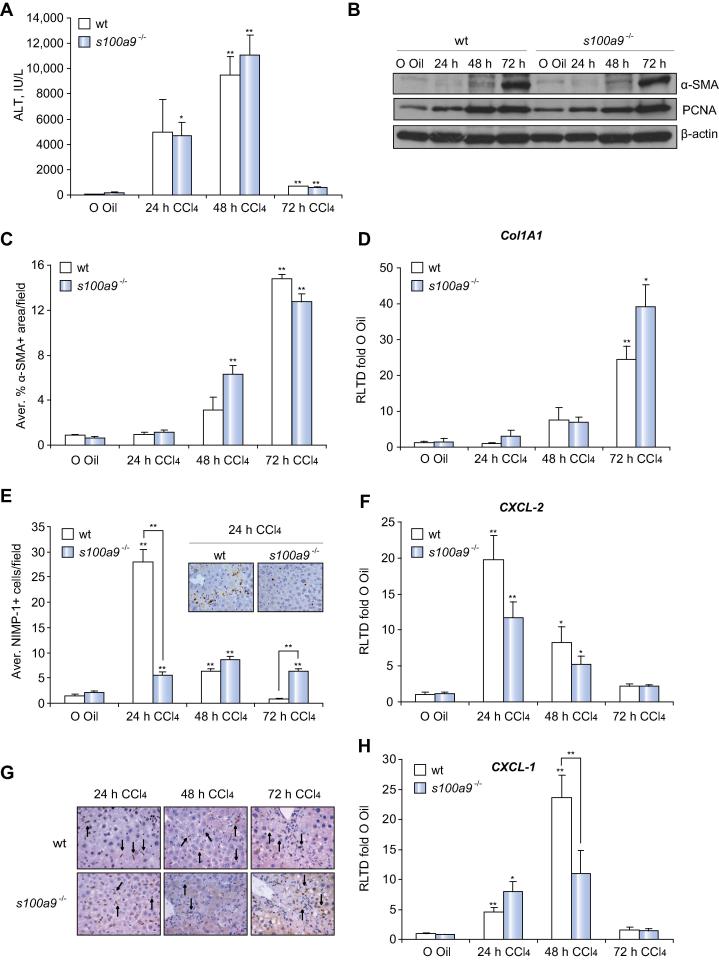
**S100A9 is required for an effective neutrophil recruitment and hepatic expression of CXCL-2.** (A) Serum analysis of ALT levels, (B) α-SMA, PCNA and β-actin western blot of whole liver lysates, (C) morphometric analysis of α-SMA+ area/field, (D) *Col1A1* mRNA expression in whole liver, (E) average NIMP-1+ cells/field with representative pictures at 400×, (F) *CXCL-2* mRNA expression, (G) CXCL-2 representative IHC pictures at 400×, cytosolic macrophage staining (black arrows), and (H) *CXCL-1* mRNA expression in whole liver of wt and *s100a9^−/−^* mice after acute CCl_4_ treatment for 24, 48, 72 h. *^∗^p* ⩽0.05; *^∗∗^p* ⩽0.01.

As Calprotectin is induced by CCl_4_ we were interested to determine a role in the hepatic wound healing response. However, absence of S100A9 made no impact on CCl_4_-induced liver damage (Fig. 3A) or the fibrogenic response as determined by hepatic αSMA protein expression (Fig. 3B), morphometry of αSMA+, and *Collagen I* gene expression (Fig. 3C–D). Normal induction of *PCNA* expression in *s100a9^−/−^* livers suggested no major role for S100A9 in the regenerative response (Fig. 3B). However, CCl_4_-induced neutrophil recruitment was defective in *s100a9^−/−^* livers (Fig. 3E). This phenotype was associated with a trend towards reduced expression of *CXCL-2* at 24 h (Fig. 3F), which by IHC staining was mainly expressed in macrophages (Fig. 3G). *CXCL-1* was also reduced at the later time point of 48 h (Fig. 3H) and was expressed in hepatocytes and macrophages (Supplementary Fig. 6A). These data suggest that both TLR2 and Calprotectin function in the recruitment of hepatic neutrophils and are required for optimal induction of CXCL-2. Given this signalling cross-talk, we determined if hepatic *TLR2* expression is influenced by S100A8/S100A9. As shown in Supplementary Fig. 6B, we observed no impact of *S100A9* knockout on hepatic TLR2 transcript expression in control uninjured (olive oil) or 24 h injured mice, this suggests that Calprotectin does not operate upstream of TLR2 in controlling neutrophil recruitment. However, we did observe a substantial 10-fold induction of TLR2 transcript at 48 h in wt liver, which was completely absent in *s100a9^−/−^* mice. We conclude that at the whole tissue level there is likely to be complex time-dependent, two-way signalling cross-talk between TLR2 and Calprotectin.

### Defective neutrophil recruitment and CXCL-2 expression do not impact on wound-healing in chronic liver disease

**Fig. 4:**
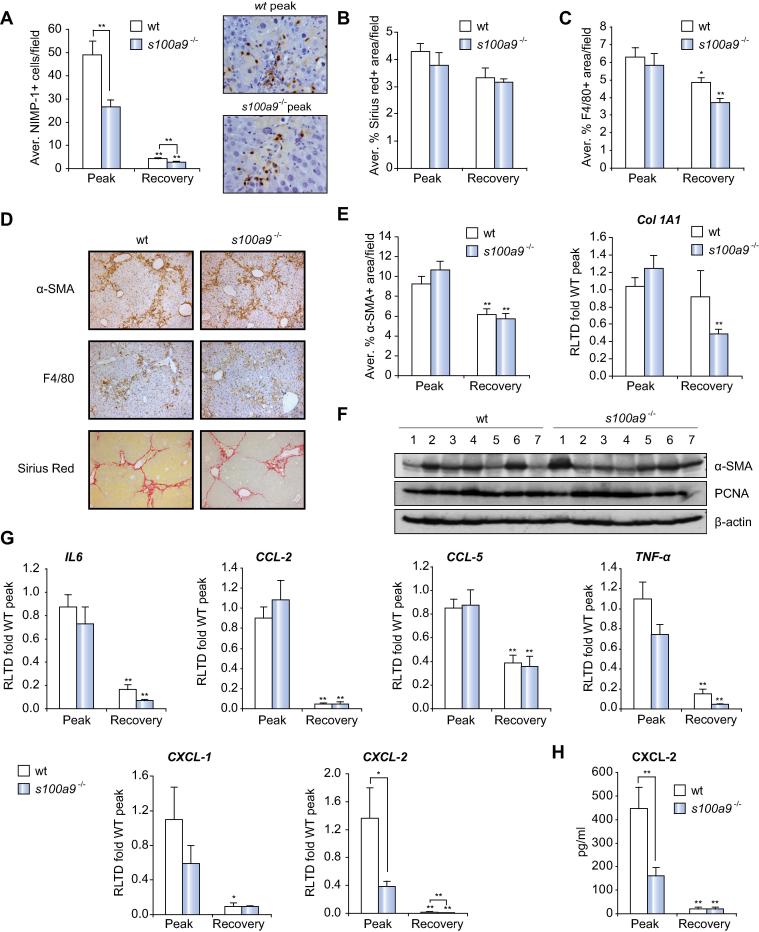
***s100a9*^−/−^ present a normal wound-healing response despite defective neutrophil recruitment and CXCL-2 expression.** (A) Average NIMP-1+ cells/field with representative pictures of peak time point at 400×, morphometric analysis of (B) Sirius Red or (C) F4/80 positive area/field. (D) Representative pictures at 100× of α-SMA, F4/80 and Sirius Red staining from peak group of wt and *s100a9^−/−^* mice. (E) Morphometric analysis of α-SMA+ area/field and *Col1A1* mRNA expression. (F) α-SMA, PCNA and β-actin western blot of whole liver from peak group of wt and *s100a9^−/−^*. (G) *IL6*, *CCL-2*, *CCL-5*, *TNF-α*, *CXCL-1* and *CXCL-2* mRNA expression. (H) CXCL-2 ELISA in whole liver of 8 week CCl_4_ treated wt and *s100a9^−/−^* mice. *^∗^p* ⩽0.05; *^∗∗^p* ⩽0.01.

To determine the impact of suppressed neutrophil recruitment in a more complex model of chronic liver disease we investigated the effects of *S100A9* deletion on wound-repair and regeneration in an 8-week model of iterative CCl_4_-induced liver disease. Two end-points were selected; 1-day (peak disease) and 7-day (recovery) post-final CCl_4_ administration, this to allow us to determine effects of *S100A9* deletion on wound healing and subsequent spontaneous repair and regeneration following cessation of injury. As anticipated we observed high numbers of hepatic neutrophils in wt mice at peak injury that dramatically declined with recovery (Fig. 4A). Numbers of neutrophils at peak injury in *s100a9^−/−^* livers were at roughly 50% of the levels found in wt, again these declined to base-line with recovery. Despite this reduced level of hepatic neutrophils we observed no differences in collagen deposition by Sirius Red staining (Fig. 4B and D), numbers of hepatic macrophages by F4/80 IHC (Fig. 4C and D), numbers of αSMA+ myofibroblasts (Fig. 4D and E) or expression of *collagen I* mRNA and αSMA protein (Fig. 4E and F). In addition, we detected similar levels of PCNA between wt and *s100a9^−/−^* livers (Fig. 4F). qRT-PCR profiling (Fig. 4G) revealed high levels of cytokines (*IL6*, *TNF-α*) and chemokines (*CCL-2*, *CCL-5*, *CXCL-1*, and *CXCL-2*) at peak disease, all of which declined to low base-line levels with recovery. In *s100a9^−/−^* we observed significantly reduced levels of CXCL-2 compared with wt, this in agreement with our earlier observation of reduced chemokine expression in acute injury (Fig. 3F). Additionally a trend towards lower expression of the neutrophil attractants *TNF-α* and *CXCL-1* was also noted in the absence of S100A9. ELISA measurements confirmed the induction of CXCL-2 and the requirement of s100a9 for this response (Fig. 4H). BDL-induced fibrosis was also relatively unaffected in s100a9^−/−^ mice (Supplementary Fig. 7A–D) although we did observe a non-significant trend towards reduced levels of collagen deposition. These data suggest that in chronic liver disease targeting neutrophil recruitment via the TLR2/Calprotectin/CXCL-2 pathway would have minimal impact on efficiency of wound-repair and regeneration.

## Discussion

Neutrophils are recruited to the hepatic sinusoids in acute liver injury and then migrate into the hepatic parenchyma in response to macrophage-derived CXC chemokines and other immune mediators released from dying/dead hepatocytes [21]. In alcoholic liver disease, APAP-induced acute liver injury and during ischemia-reperfusion, activated neutrophils within the parenchyma are potentially harmful as they can promote hepatocellular stress and necrosis contributing to liver failure [2]. Hence, illuminating the molecular regulators of neutrophil recruitment and extravasation is of interest for a better understanding of hepatic immunity and for developing strategies aimed at limiting collateral tissue damage caused by activated neutrophils. Here we report that TLR2 and S100A9 are required in a non-redundant manner for optimal induction of hepatic CXCL-2 and recruitment of neutrophils in response to hepatocellular damage. The expression of TLR2 has been detected on a number of different resident liver cell types including Kupffer cells, hepatic stellate cells, hepatocytes, cholangiocytes and sinusoidal endothelial cells [22]. In previous studies by the Seki lab employing TLR2 bone marrow chimeric mice, Kupffer cells were found to be the dominant cell type through which hepatic inflammation is mediated by TLR2 [23]. Most likely from our IHC studies CXCL-2 is chiefly induced within Kupffer cells via intracellular TLR2 signalling with more modest, possibly secondary expression appearing in damaged hepatocytes, which would be in agreement with other reports in the literature [24,25]. However the cellular source of CXCL-2 may be dependent on the nature of the liver injury. Xu *et al.* showed that α-Napthylisothiocyanate (ANIT), which causes severe cholestatic injury in the mouse was associated with rapid induction of CXCL-2 primarily from periportal hepatocytes [26]. Although, of note, the authors observed only a modest induction of CXCL-2 in cultured hepatocytes directly exposed to ANIT [26]. S100A9 is mainly associated with granulocytes, but was induced along with S100A8 in hepatocytes following liver damage. This observation is in keeping with others who have documented inducible expression of *S100A8* and *S100A9* in epithelial cells of injured tissues [14]. Furthermore, *S100A8* and *S100A9* are expressed by HCC tumour cells as well as by other tumours of epithelial origin including lung, breast, gastric, and prostate [27]. Hence, we propose that dual signalling via TLR2 on Kupffer cells and S100A8/S100A9 from hepatocytes combine to generate CXCL-2 chemokine gradients for guidance of neutrophils into the hepatic sinusoids and parenchyma respectively.

Marques *et al.* recently showed that CXCR2 antagonism in mice injured with APAP suppressed hepatic neutrophil recruitment by 50% [28]. In this latter study combined antagonism of CXCR2 and FPR1 resulted in more substantive suppression of neutrophil migration; this is in agreement with McDonald *et al.*, who reported a cooperation between CXCR2 chemokines and mitochondrial formyl peptides for guidance of neutrophils to sites of necrosis [29]. In the ANIT cholestasis model a 50% reduction in the influx of neutrophils was observed in *cxcr2^−/−^* mice [26], which closely agrees with data from Marques *et al.* when employing a pharmacological approach for blockade of CXCR2 in the APAP model. Hence, CXCR2 chemokines are not absolutely required for neutrophil influx to injured liver, but instead cooperate with other neutrophil attractants such as ATP, formyl peptides and TNF-α to ensure optimal neutrophil guidance [11]. Our data build on these findings by revealing that TLR2 and S100A8/S100A9 operate in neutrophil guidance most likely upstream of CXCL-2 (and to a lesser extent CXCL-1 and TNF-α) by regulating its expression in response to tissue damage. Signalling pathways downstream of TLR2 and S100A8/S100A9 both converge on activation of NF-κB, for which *CXCL-1* and *CXCL-2* are known target genes [30], this providing one plausible explanation for the similar regulatory functions of TLR2 and S100A8/S100A9 in neutrophil recruitment.

A key finding of our work was that acute repair and regenerative responses were normal in *TLR2* and *S100A9* knockout mice, and furthermore fibrosis caused by chronic injury with CCl_4_ was unaffected in *S100A9* knockouts. Previous studies have shown little or no influence of neutrophilic inflammation on fibrosis [9], in addition ANIT-induced fibrosis is unaffected in *cxcr2^−/−^* animals [26]. Hence, our data are supportive of neutrophils and CXCR2 chemokines being redundant for fibrogenesis. The role of TLR2 in fibrosis is unclear since there are apparently contradictory data in the recent literature. Seki *et al.*, observed that while *tlr4^−/−^* mice are attenuated for liver fibrosis induced by CCl_4_ and BDL, by contrast *tlr2^−/−^* developed BDL-induced fibrosis in a similar manner to wt mice [31]. However, in subsequent studies Hartmann and colleagues found that TLR2 deficient mice re-derived and housed in specific pathogen-free (sp-f) conditions were resistant to BDL- and CCl_4_-induced fibrosis [32]. In the latter study, it was suggested that TNF-α produced by TLR2+ monocytes in the intestinal lamina propria mediates intestinal barrier disruption, resulting in translocation of bacteria and their products across the mucosal barrier and on to the liver via the portal circulation, where they enhance fibrogenesis. The Seki lab also recently reported that *tlr2^−/−^* mice are protected from progression of CDAA-induced NASH to fibrosis and they suggested this was due to an impaired inflammatory reaction associated with reduced expression of NLRP3 inflammasome components [23]. Hence, neither of these latter studies suggest a direct role for TLR2 in hepatic stellate cell activation, but instead indicate that absence of TLR2 results in failure of inflammatory pathways that are upstream of fibrosis. Our observation of normal induction of *α-SMA* and *collagen I* expression in *tlr2^−/−^* following acute injury with CCl_4_ argues against a direct role for TLR2 in hepatic stellate cell activation.

In summary we have advanced current knowledge regarding the mechanisms by which neutrophils are guided to the injured liver and identified TLR2 and S100A8/S100A9 as key regulators of hepatic CXCL-2 expression and neutrophil recruitment. This new information may be of use in developing strategies for limiting neutrophil-mediated tissue damage in acute liver injuries.

## Conflict of interest

The authors who have taken part in this study declared that they do not have anything to disclose regarding funding or conflict of interest with respect to this manuscript.
