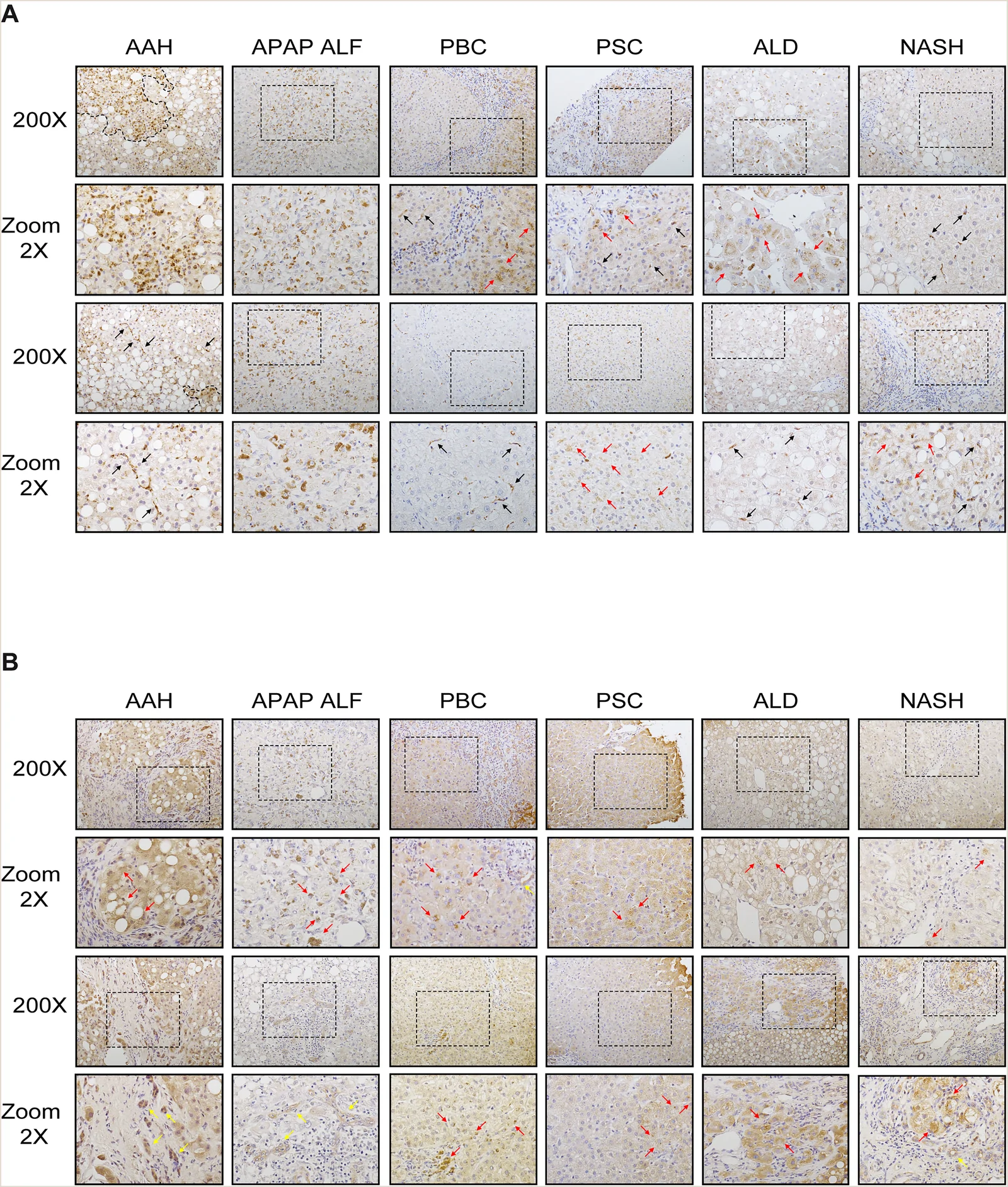# Corrigendum to: “A TLR2/S100A9/CXCL-2 signaling network is necessary for neutrophil recruitment in acute and chronic liver injury in the mouse” [J Hepatol (2014) 60: 782-91]

**DOI:** 10.1016/j.jhep.2026.06.017

**Published:** 2026-09

**Authors:** Anna Moles, Lindsay Murphy, Caroline L. Wilson, Jayashree Bagchi Chakraborty, Christopher Fox, Eek Joong Park, Jelena Mann, Fiona Oakley, Rachel Howarth, John Brain, Steven Masson, Michael Karin, Ekihiro Seki, Derek Mann

**Affiliations:** 1 Fibrosis Research Group, Institute of Cellular Medicine, Newcastle University, Newcastle Upon Tyne, UK; 2 Division of Gastroenterology, Department of Medicine, University of California, San Diego, School of Medicine, La Jolla, California, USA; 3 Departments of Pharmacology and Pathology, Laboratory of Gene Regulation and Signal Transduction, School of Medicine, University of California, San Diego, 9500 Gilman Drive, La Jolla, CA 92093-0723, USA

It has been brought to our attention via PubPeer that an error is present in Supplementary Fig. 4A in our paper.

Specifically, the panel showing CXCL1 staining in patients with APAP ALF incorrectly included duplicate representative images from a single patient at ×200 magnification, rather than images from two distinct patients as intended. As a consequence, the corresponding zoomed regions of interest were also derived from overlapping areas of the same image.

This error arose during figure assembly and does not affect the conclusions of the study. The figure has now been corrected using the original time-stamped CXCL1 staining images from two separate patients with APAP ALF (patient codes 16290 and 40170), ensuring that representative images from both individuals are shown.

The corrected Supplementary Fig. 4A is provided below.

**Figure:**