# Supplementary material for: A TLR2/S100A9/CXCL-2 signaling network is necessary for neutrophil recruitment in acute and chronic liver injury in the mouse
Source: J Hepatol. 2014 Apr;60(4):782–91. doi: 10.1016/j.jhep.2013.12.005 (PMC3960359; doi:10.1016/j.jhep.2013.12.005)
Supplement: Supplementary Figs. 1–7 [file mmc1.pdf]

Supplementary Figures

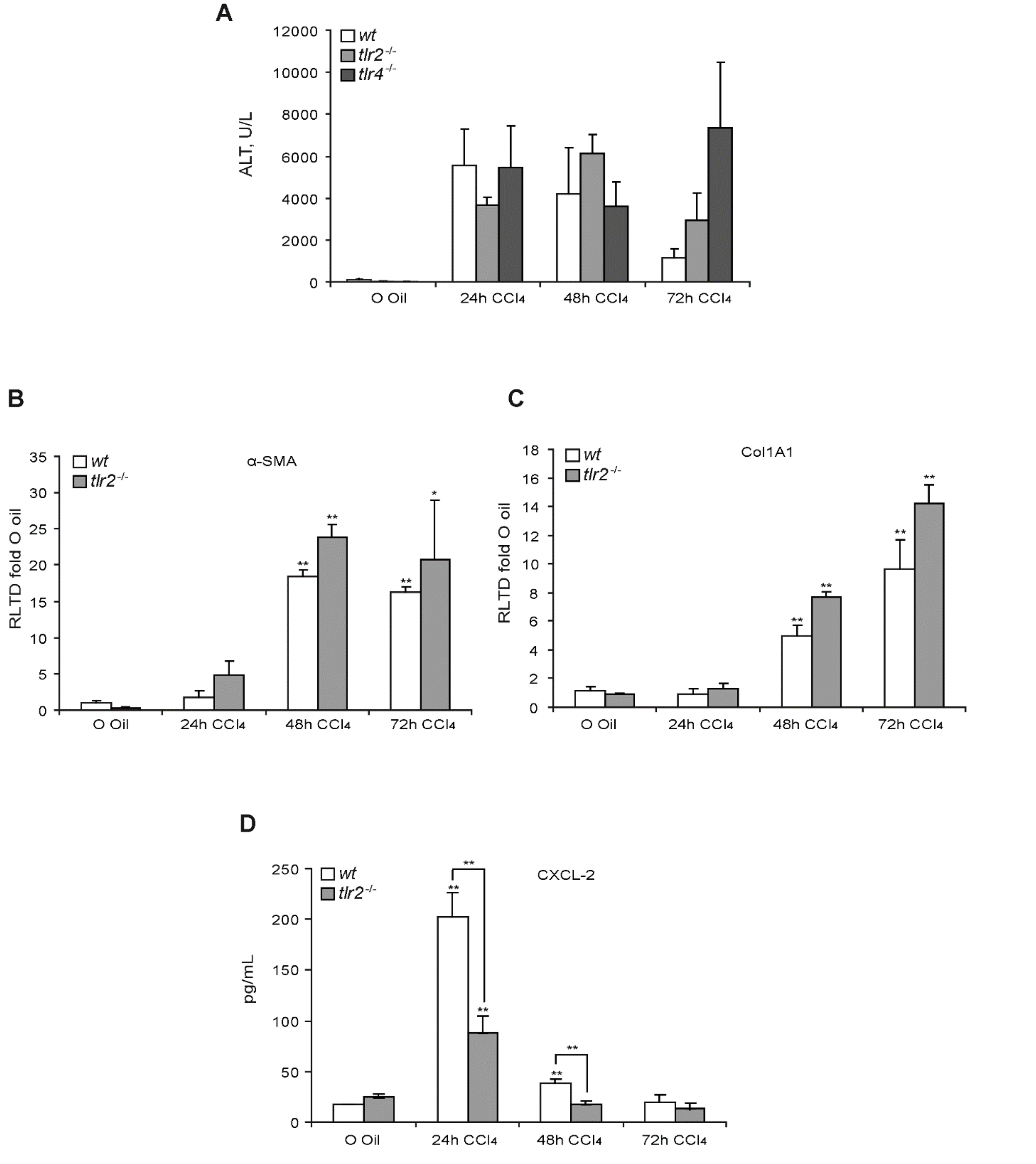

Supplementary Fig. 1. Liver damage and fibrogenic gene expression in acute CCl_4 injury in *wt*, *tlr2*$^{-/-}$ and *tlr4*$^{-/-}$. Serum analysis of (A) ALT levels, (B) α-SMA, and (C) Collagen 1A1 (Col1A1) mRNA expression. (D) CXCL-2 ELISA in whole liver in *wt*, *tlr2*$^{-/-}$ and *tlr4*$^{-/-}$ mice after acute CCl_4 treatment for 24, 48, 72 h.

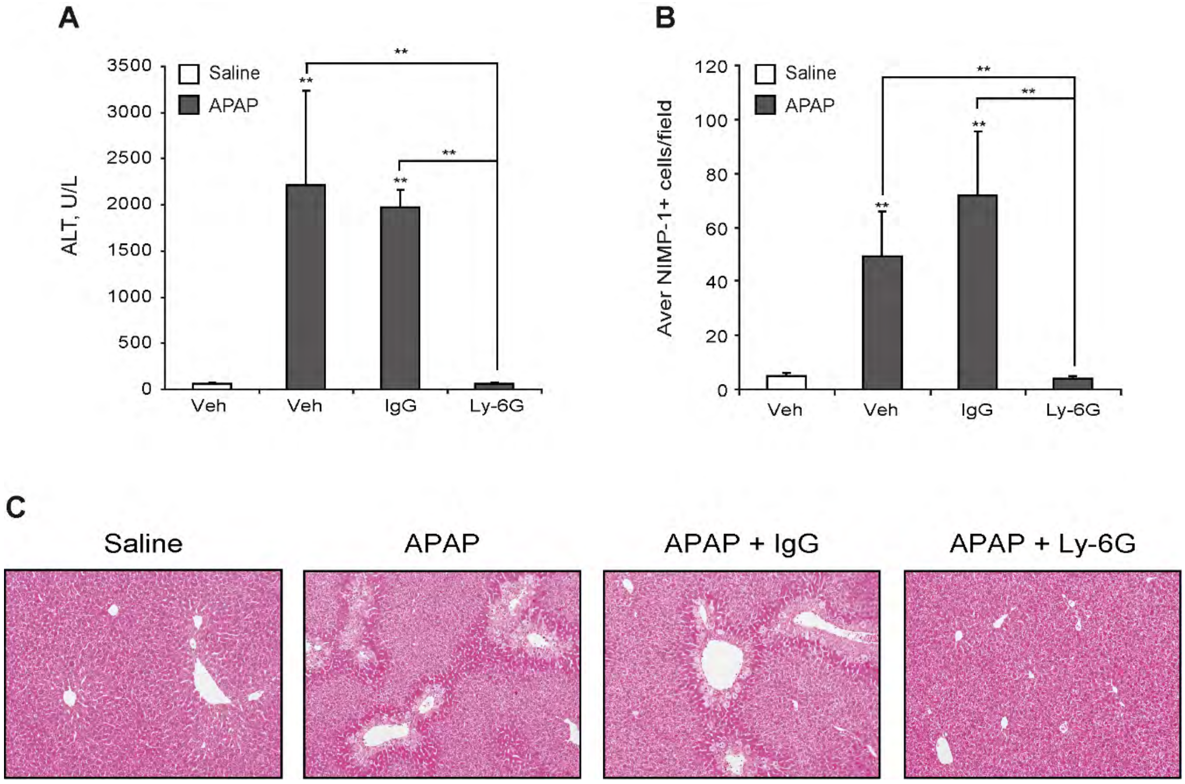

Supplementary Fig. 2. Role of neutrophils in APAP mouse model. (A) Serum analysis of ALT levels, (B) average NIMP-1+ cells/field, (C) H&E representative pictures at 100X, in *wt* pre-treated with Ly-6G or IgG for 12 h and afterwards treated with APAP or saline for 24 h.

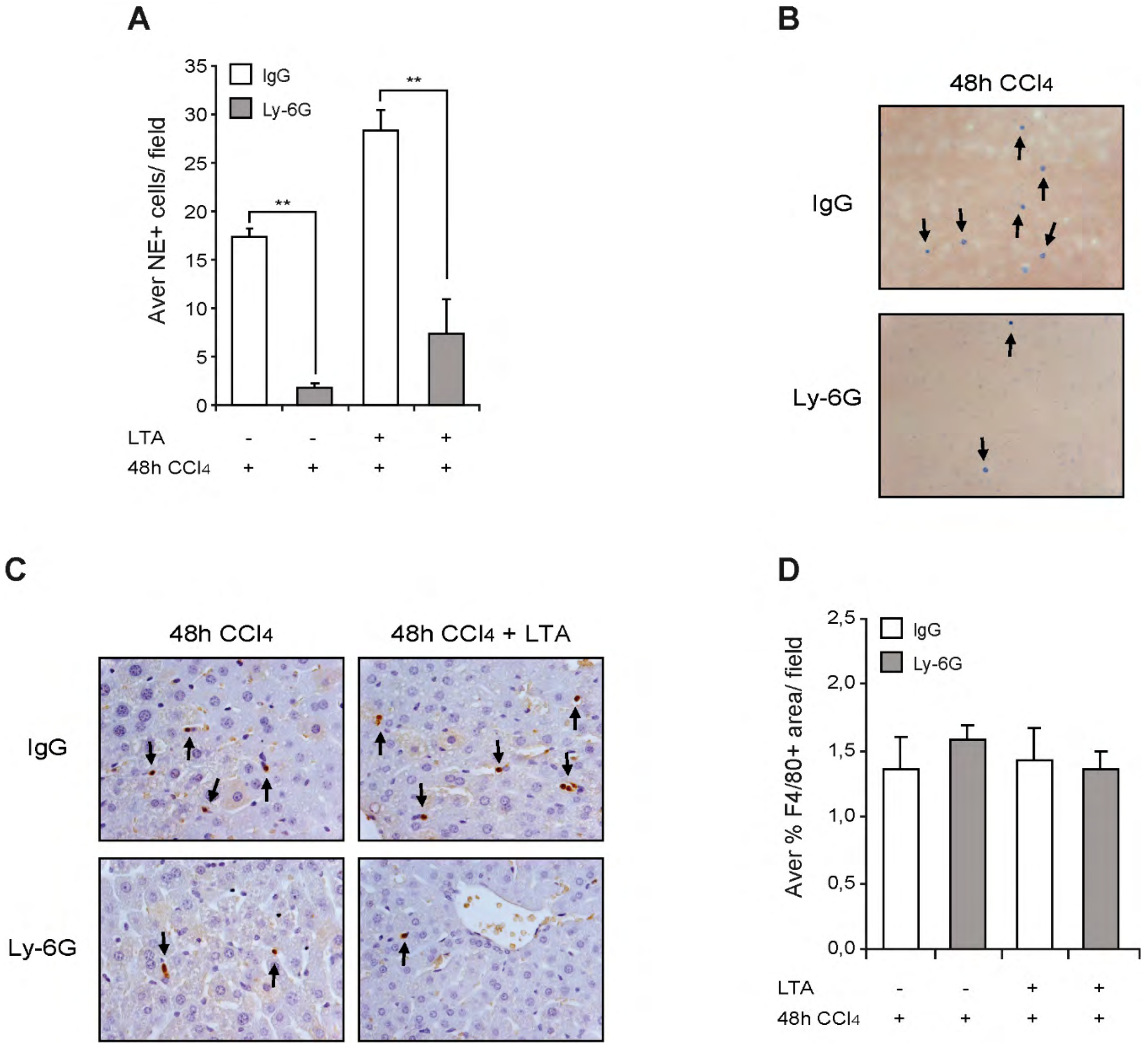

Supplementary Fig. 3. Efficiency of neutrophil depletion by Ly-6G antibody. (A) Average Neutrophil elastase (NE)+ cells/field, (B) representative pictures of Naftol staining in blood smear at 400X, (C) NIMP-1 IHC representative pictures at 400X, (D) morphometric analysis of F4/80+ area in *wt* pre-treated with Ly-6G or IgG for 12 h and afterwards treated with CCl_4 for 48 h ± LTA.

A

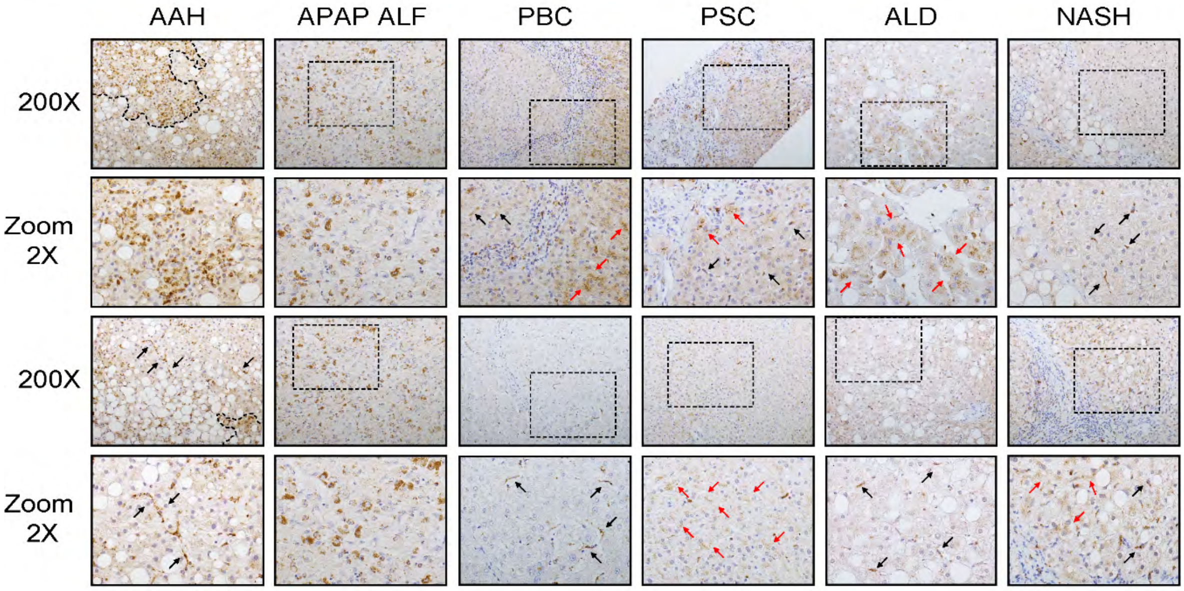

B

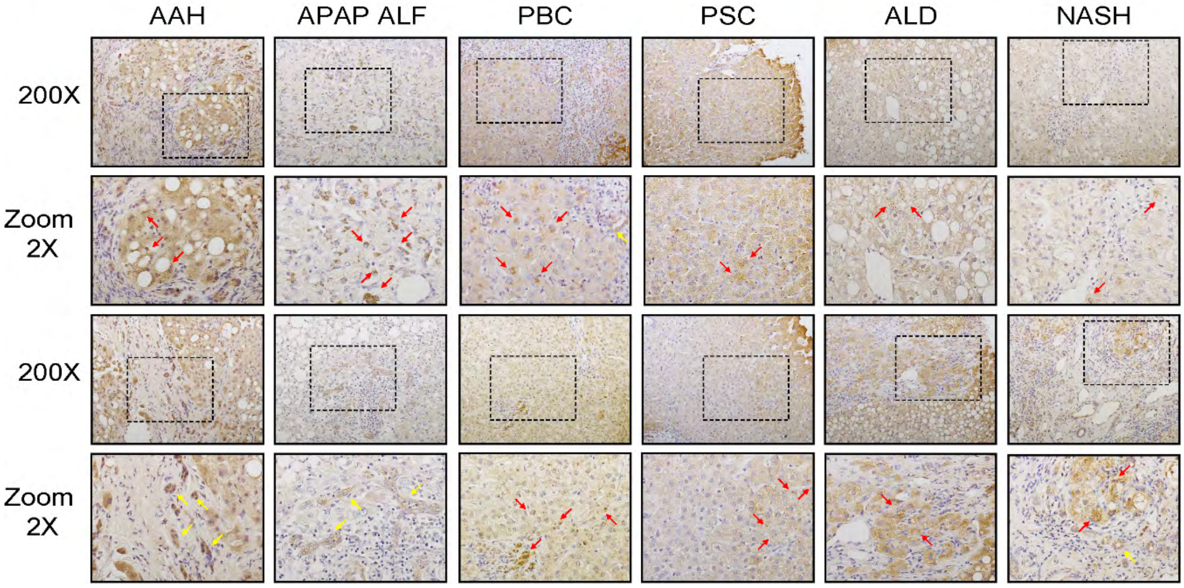

Supplementary Fig. 4. CXCL-1 and CXCL-2 expression in acute and chronic human liver diseases. Representative pictures of two different patients at 200X and zoom 2X of (A) CXCL-1 and (B) CXCL-2 from a minimum of 4 different human biopsies of AAH, APAP ALF, PBC, PSC, ALD, and NASH. Black arrows indicate macrophages, red hepatocytes and yellow bilary epithelial cells.

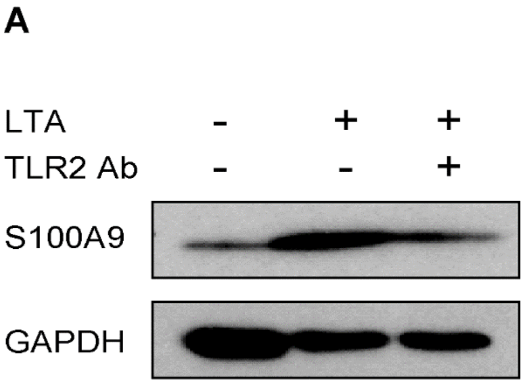

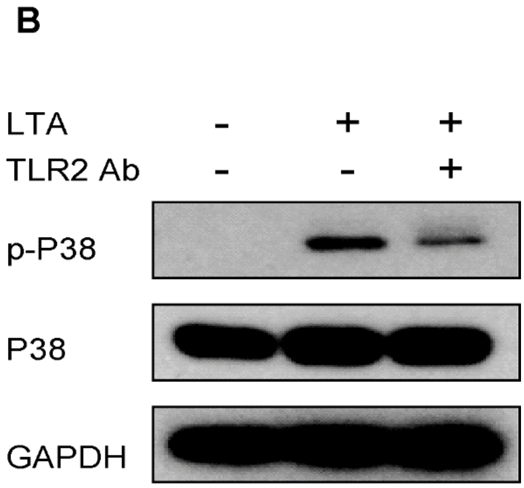

Supplementary Fig. 5. S100A9 expression in *in vitro* cultured neutrophils is TLR2 dependent. (A) S100A9 and GAPDH, (B) pP38, total P38 and GAPDH western blots from neutrophil lysates. Neutrophils were pre-treated with anti-mTLR2-IgG for 1 h at 2μg/mL before stimulation with LTA at 10μg/mL for 45 min.

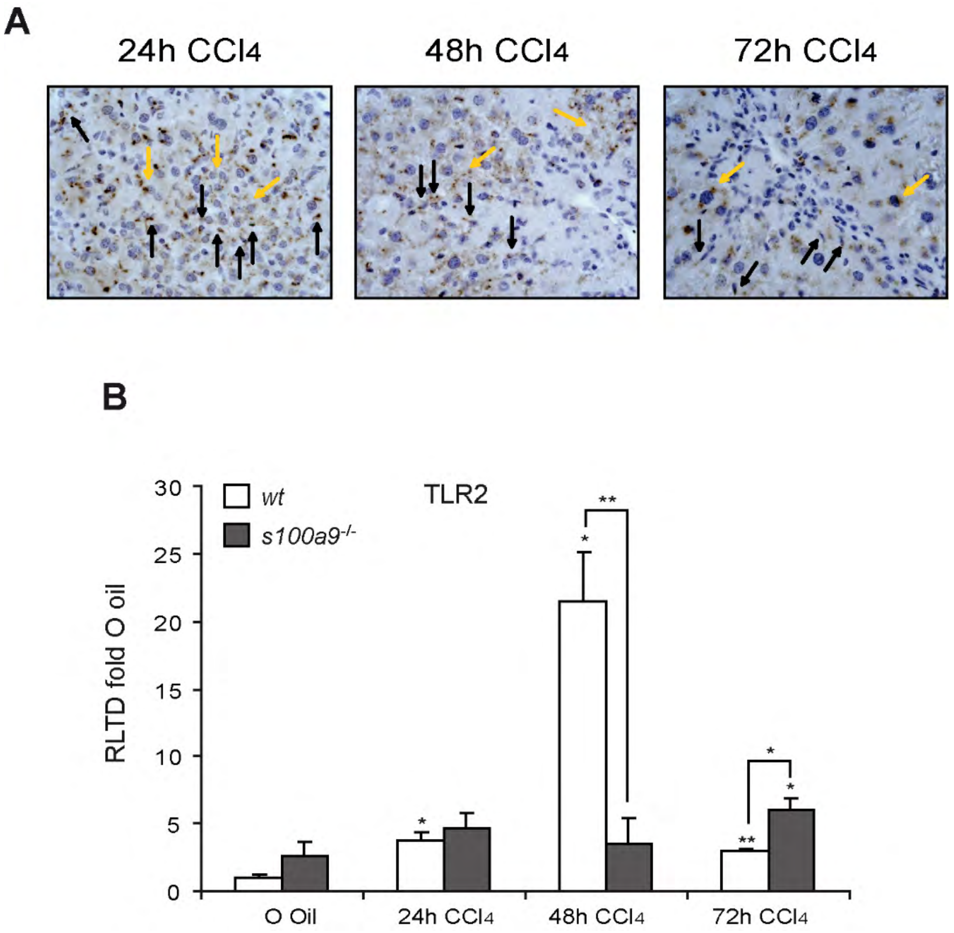

Supplementary Fig. 6. CXCL-1 and TLR2 expression in *s100a9*$^{-/-}$ after acute CCl_4 injury. (A) CXCL-1 representative IHC 400X pictures, cytosolic macrophage staining (black arrows), cytosolic hepatocyte staining (yellow arrows), (B) *TLR2* mRNA expression of *wt* and *s100a9*$^{-/-}$ after acute CCl_4 treatment for 24, 48, 72 h.

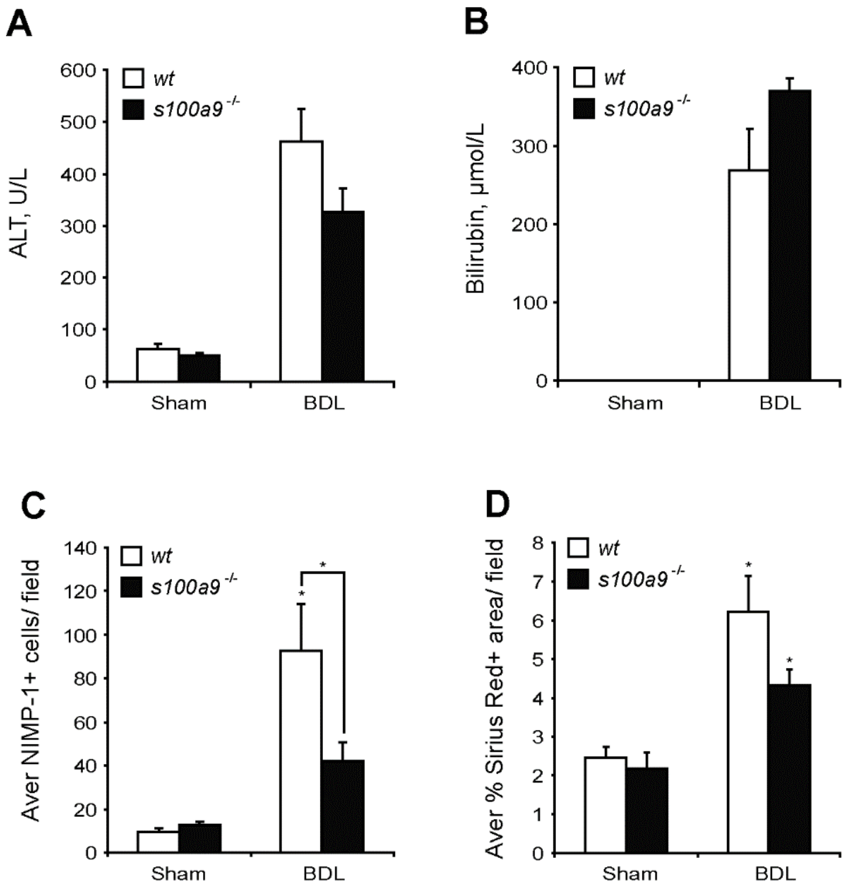

Supplementary Fig. 7. *S100a9*$^{-/-}$ mice present a normal wound-healing response after 14 days bile duct ligation (BDL) despite defective neutrophil recruitment. (A) Serum analysis of ALT levels and (B) bilirubin levels. (C) Average NIMP-1+ cells/field, (D) morphometric analysis of Sirius Red positive area/field, in *wt* and *s100a9*$^{-/-}$ after sham or 14 days BDL surgery.
