# Supplementary material for: A TLR2/S100A9/CXCL-2 signaling network is necessary for neutrophil recruitment in acute and chronic liver injury in the mouse
Source: J Hepatol. 2014 Apr;60(4):782–91. doi: 10.1016/j.jhep.2013.12.005 (PMC3960359; doi:10.1016/j.jhep.2013.12.005)
Supplement: Supplementary Materials and methods [file mmc2.pdf]

Supplementary Materials and methods

Reagents

Carbon tetrachloride and olive oil were purchased from Sigma-Aldrich. Purified lipoteichoic acid (LTA) from *Staphylococcus aureus* and anti-mTLR2-IgG were from Invivogen. Purified anti-mouse Ly-6G and rat IgG2a isotype control were from Biolegend. Avidin/Biotin blocking kit, citric acid based Antigen Unmasking Solution, Vectastain Elite ABC Reagent and DAB peroxidase substrate kit were purchased from Vector Laboratories. Formalin and Mayer's Haematoxylin were from Sigma-Aldrich. CXCL-2/MIP-2 Quantikine ELISA kit was from R&D Systems. TRI reagent and SYBR Green JumpStart Taq ready mix were from Sigma-Aldrich. Reagents for cDNA synthesis were from Promega. Unless otherwise reported all other reagents were from Sigma-Aldrich.

In-vivo model of rodent acute liver injury by acetaminophen administration

12 weeks old male C57Bl/6 littermate mice were injected IP with Ly-6G or IgG control antibody 12 hours before injection of acetaminophen at 350mg/Kg. Animals were culled 24 hrs after the acetaminophen injection. At least 7 animals were used per group.

RNA isolation and real time PCR

Total RNA was isolated from liver tissue using TRI reagent. RNA was precipitated with isopropanol and washed with 70% alcohol before being measured by Nanodrop. cDNA synthesis was performed using Promega kit. Real time PCR was performed with SYBR Green JumpStart Taq ready mix following the manufacturer's instructions. Primer sequences used are reported in the supplementary methods.

Immunohistochemistry

Immunohistochemistry was performed on 4μm liver sections. αSMA, NIMP-1, F4/80 [1], and PCNA [18] were performed as previously described. Neutrophil elastase (Abcam) stain was performed as NIMP-1. For CXCL-2 (2b scientific) and S100A9 (Abcam), deparaffinised sections were incubated in hydrogen peroxide/methanol. Antigen retrieval was achieved by Proteinase K (20μg/mL) or Antigen Unmasking Solution respectively. Endogenous avidin and biotin were blocked. Then 20% swine serum was added. Sections were incubated overnight with primary antibodies and developed and counterstain with Mayer's haematoxylin next day. For NIMP-1, neutrophil elastase and PCNA slides were blinded and a minimum of 15 random fields at 200X were manually counted. Image analysis was performed at 100X using Leica Qwin for αSMA, and F4/80 stainings. AAH, APAP ALF, PBC, PSC, ALD and NASH liver samples were taken under full ethical approval with patient consent (REC references 10/H0903/32, 10/H0906/41, 06/Q0905/150, and 10/H0903/32), at least 10 random fields at 100X were manually counted for the neutrophil elastase staining in human biopsies.

H&E and Sirius Red staining

Livers were fixed with formalin, embedded in paraffin, and sections of 4μm were routinely stained with H&E or 0.1% Sirius Red following standard procedures.

SDS-PAGE and immunoblotting

Total protein liver lysates were prepared in radioimmune precipitation assay buffer (RIPA). Samples containing 10-30μg of protein were fractionated by 8-10% SDS-PAGE and transferred to nitrocellulose. Blots were blocked with TBS/Tween 20 (0.1%, T-TBS) containing 5% nonfat milk or BSA before overnight incubation with primary antibodies

anti-α-SMA and anti-β-actin (Sigma-Aldrich, 1:1000), anti-PCNA, anti-S100A9, anti-GAPDH (Abcam, 1:250, 1:1000; 1:1000), anti-pP38 and p38 (Cell Signaling, 1:1000). Membranes were washed in T-TBS, incubated with secondary HRP-conjugated antibodies and developed by ECL (Thermo Scientific).

Neutrophil isolation and treatment

Neutrophils were isolated from the bone marrow of eighteen C57Bl/6 mice. Briefly, the bone marrow was flush with Hank´s Balanced Salt Solution (HBSS) from the femur and the tibia. The red blood cells were lysed with NaCl and then cells were passed through a 100 µm cell restrainer and centrifuged at 400g for 5 minutes. Pellet was resuspended in HBSS, loaded in a 63% Percoll gradient and centrifuged at 1000g for 30 minutes. Neutrophils where collected and wash twice at 400g for 5 minutes and kept in culture with RPMI media supplemented with 10% FBS. Neutrophils where pre-treated with anti-mTLR2-IgG for 1hr at 2µg/mL before stimulation with LTA at 10µg/mL for 45 minutes and then lysate with RIPA buffer.

CXCL-2 enzyme-linked immunosorbent assay (ELISA)

Whole liver samples were lysate with 100mM Tris (pH: 7.4), 150mM NaCl, 1mM EDTA, 1mM EGTA, 1% Triton X-100, 0.5% sodium deoxycholate. ELISA was performed according to manufacturer's instructions.

Supplementary Table 1. Mouse primer sequences.

Gene (GenBank Accession)	Primer sequence
GAPDH (NM_008084)	Fw: 5’-GCACAGTCAAGGCCGAGAAT-3’
	Rv: 5’-GCCTTCTCCATGGTGGTGAA-3’
α-SMA (NM_007392)	Fw: 5’-TCAGCGCCTCCAGTTCCT-3’
	Rv: 5’-AAAAAAAACCACGTAACAAATCAA-3’
Collagen 1A1 (NM_007742)	Fw: 5’-TTCACCTACAGCACGCTTGTG-3’
	Rv: 5’-GATGACTGTCTTGCCCCAAGTT-3’
CXCL-1 (NM_008176)	Fw: 5’-CTGGGATTCACCTCAAGAACATC-3’
	Rv: 5’-CAGGGTCAAGGCAAGCCTC-3’
CXCL-2 (NM_009140)	Fw: 5’-CCAACCACCAGGCTACAGG-3’
	Rv: 5’-GCGTCACACTCAAGCTCTG-3’
TNF-α (NM_013693)	Fw: 5’-CCCTCACACTCAGATCATCTTCT-3’
	Rv: 5’-GCTACGACGTGGGCTACAG-3’
IL-6 (NM_031168)	Fw: 5’-TAGTCCTTCCTACCCCAATTTCC-3’
	Rv: 5’-TTGGTCCTTAGCCACTCCTTC-3’
CCL-2 (NM_013693)	Fw: 5’-AGGTCCCTGTCATGCTTCTG-3’
	Rv: 5’-TCTGGACCCATTCCTTCTTG-3’
CCL-5 (NM_031168)	Fw: 5’-TGCTGCTTTGCCTACCTCTCC-3’
	Rv: 5’-TGGCACACACTTGGCGGTTCC-3’
S100A8 (NM_013650)	Fw: 5’-TGCGATGGTGATAAAGTGG -3’
	Rv: 5’-GGCCAGAAGCTCTGCTACTC-3’
S100A9 (NM_009114)	Fw: 5’-CACCCTGAGCAAGAAGGAAT -3’
	Rv: 5’-TGTCATTTATGAGGGCTTCATTT-3’
TLR2 (NM_011905)	Fw: 5’- GCAAACGCTGTTCTGCTCAG -3’
	Rv: 5’- AGGCGTCTCCCTCTATTGTATT -3’

Reference

[1] Moles A, Sanchez AM, Banks PS, Murphy LB, Luli S, Borthwick L, et al. Inhibition of RelA-Ser536 phosphorylation by a competing peptide reduces mouse liver fibrosis without blocking the innate immune response. Hepatology 2013;57:817—828.
